# A Comparative Analysis of Follicular Diameter Assessment Versus Doppler Ultrasound in Predicting Ovulation Timing for the Infertility Treatment: Insights From a Prospective Study

**DOI:** 10.7759/cureus.61466

**Published:** 2024-05-31

**Authors:** Rajasingh Revathi, Abi Chandrasekaran, Dhinaharan Pookanraj, Janani Moorthy

**Affiliations:** 1 Department of Obstetrics and Gynaecology, Bhaarath Medical College and Hospital, Chennai, IND; 2 Department of Urology, Bhaarath Medical College and Hospital, Chennai, IND; 3 Department of Radiodiagnosis, ACS Medical College and Hospital, Chennai, IND

**Keywords:** infertility therapy, infertility treatment, doppler ultrasound, follicular diameter, ovulation timing prediction

## Abstract

Background

Infertility remains a significant challenge affecting millions of couples worldwide, with ovulation abnormalities being a common underlying cause. Pharmacological methods, such as clomiphene citrate, are often used to stimulate ovulation. However, the optimal timing for sexual intercourse during ovulation induction remains contentious.

Objectives

This study aimed to compare the efficacy of transvaginal ultrasonography (TVS) for measuring follicle size with Doppler ultrasound for assessing changes in blood flow to predict the timing of ovulation.

Methods

We conducted a comparative analysis involving 64 women undergoing infertility therapy. Participants were evaluated using both TVS to measure follicle diameter and Doppler ultrasound to assess perifollicular blood flow dynamics. The primary outcomes measured included ovulation rates, resistive index (RI) values, peak systolic velocity (PSV) values, and conception rates.

Results

The analysis showed comparable age distributions between the TVS and Doppler groups. There was no significant correlation between follicle diameter and ovulation when assessed by TVS. However, Doppler ultrasound revealed a substantial association between perifollicular blood flow dynamics and ovulation. Higher ovulation rates were linked to lower RI values and higher PSV values, indicating their potential as predictors of ovulation. Additionally, higher conception rates were positively correlated with increased vascularity in Zone 4 of the endometrium.

Conclusion

Doppler ultrasonography indices, particularly RI and PSV values, provide critical insights into perifollicular blood flow dynamics and endometrial vascularity, which can enhance the effectiveness of fertility treatments. While these findings highlight the potential of Doppler ultrasound in predicting ovulation and improving treatment outcomes, further research is required to understand the underlying mechanisms and validate these results for personalised treatment strategies.

## Introduction

Infertility, characterised by one year of unprotected intercourse without conception, affects approximately 10% of couples worldwide, with female-related issues contributing significantly [1]. Among these, disorders of ovulation account for 30% to 40% of female infertility cases [2], often presenting as common causes of subfertility and reproductive challenges in women of childbearing age. Pharmacological interventions aimed at inducing or augmenting ovulation offer varying rates of success, with clomiphene citrate standing out as a first-line treatment due to its cost-effectiveness and minimal side effects [3]. The mechanism of action of clomiphene citrate involves disrupting oestrogen feedback mechanisms at the pituitary and hypothalamic levels, thereby enhancing the secretion of gonadotropin-releasing hormone (GnRH) and the subsequent production of follicle-stimulating hormone (FSH) [4]. This stimulation promotes follicular growth and maturation, culminating in the emergence of one or more dominant follicles. Dosage adjustments, typically initiated at 50 mg daily for five days per cycle, may be implemented based on patient response, with ovulation anticipated within five to 10 days post-treatment [5].

Despite established protocols, the optimal timing of intercourse during ovulation induction remains a subject of debate, with suggestions ranging from frequent intercourse to timed coitus around the expected time of ovulation. Moreover, while approximately 46% of women may ovulate with a 50-mg daily dosage of clomiphene citrate, increasing the dosage to 100 mg may further enhance ovulation rates [6]. Ovulation detection forms a cornerstone in the diagnostic evaluation of subfertile couples and plays a pivotal role in family planning programmes and assisted reproductive techniques (ARTs). Although various techniques exist for ovulation detection, direct methods such as transvaginal ultrasonography (TVS) offer superior accuracy by visualising follicular growth and rupture [7].

The integration of Doppler ultrasound into TVS procedures has revolutionised follicular and endometrial assessment in ARTs. Doppler ultrasound enables the evaluation of vascular changes within the ovary and endometrium, providing insights into follicular and endometrial maturity and receptivity. These vascular dynamics serve as crucial indicators of biochemical and morphological changes associated with follicular development, ovulation, and luteinisation [8]. In the context of intrauterine insemination (IUI) combined with clomiphene citrate, the precise timing of the procedure in relation to ovulation is paramount for optimising pregnancy rates. IUI offers a less invasive and cost-effective alternative to in vitro fertilisation (IVF), albeit with increased risks of multiple pregnancies when combined with ovarian stimulation [9]. The present study endeavours to compare the efficacy of TVS-based follicular diameter (FD) assessment with Doppler ultrasound-based vascular changes in predicting ovulation timing. By elucidating the respective roles of these modalities, the study aims to refine clinical practices and enhance reproductive outcomes in couples undergoing fertility treatments.

## Materials and methods

A comparative study was conducted on 64 women who visited the Level 2 Ultrasound (USG) and Doppler Infertility Outpatient Department of Obstetrics and Gynaecology at Bhaarath Medical College and Hospital, Chennai, India, between 2022 and 2023. The purpose of the study was to investigate the effectiveness of infertility treatment, with each participant undergoing a maximum of five treatment cycles. The objective of this study was to evaluate and compare the efficacy of TVS in measuring follicle diameter, with Doppler ultrasound in assessing vascular alterations and predicting the timing of ovulation. The Ethical Review Committee of Bhaarath Medical College and Hospital, Tamil Nadu, India, granted ethical approval in 2022, with clearance number BMCH/IEC/2022/180.

Criteria for inclusion and exclusion

The present research includes patients aged 18 to 35 who were receiving ovulation induction for the treatment of anovulatory infertility. Participants were diagnosed with either primary or secondary infertility. They must have unobstructed fallopian tubes, as determined by diagnostic imaging or previous surgical results. Furthermore, these individuals were required to submit conventional semen analysis findings and hormonal markers in order to guarantee a homogeneous study group with normal reproductive baselines. Patients were eliminated from the study based on a number of factors to ensure the reliability and specificity of the findings. Endometriosis, a disorder characterised by the presence of endometrial tissue outside the uterus, was excluded since it has the potential to interfere with fertility therapies and results. Patients with organic ovarian cysts that demonstrated functional characteristics, as confirmed by ultrasonography and clinical examination, were also eliminated. Another exclusion criterion was insufficient responsiveness to ovulation induction, which ensured that only individuals who responded reliably to the medication were included in the study.

In addition, patients with uterine myomas, which are benign tumours that form in the uterus, were eliminated since they have the potential to affect implantation and pregnancy outcomes. Another exclusion criterion was fallopian tube obstruction, which might be diagnosed through imaging or by past medical history. Patients with aberrant sperm analysis or hormonal data were removed to ensure a consistent baseline for all subjects. Finally, those who had received induction regimens for more than six rounds were eliminated to prevent potential biases caused by prolonged exposure to fertility therapies.

Study designs

The study included women who were receiving infertility therapy at the Level 2 USG and Doppler Infertility Outpatient Department of Obstetrics and Gynaecology at Bhaarath Medical College and Hospital from 2022 to 2023. Patients were given comprehensive information regarding the techniques, induction protocols, and objectives of TVS and Doppler studies, and the accompanying expenses were clarified. According to this data, patients were divided into two groups. Comprehensive demographic information, such as age, weight, body mass index (BMI), and hormone markers, was gathered from both groups [10]. A baseline scan was conducted on the second day of the menstrual cycle to confirm the absence of active follicles, low levels of oestrogen and progesterone, and a thin endometrium.

Ovulation induction was commenced by administering clomiphene citrate at an initial dosage of 50 mg, with a maximum dosage of 100 mg, for five days commencing from day 5 of the menstrual cycle in both groups. Patients were directed to undergo follicular investigation every other day, beginning on day 9 of their menstrual cycle [11]. Within the TVS group [12], the size of the follicle was assessed, and an injection of human chorionic gonadotropin (hCG) was given when the follicle attained a diameter of either 15-16 mm or 17-18 mm. The following TVS was conducted on a subsequent day to verify the rupture of the follicle, and IUI was then scheduled. Vaginal progesterone pessaries were used for 10 days after IUI to give hormonal assistance during the luteal phase. In the Doppler group, alongside measuring the diameter of the follicle, a Doppler assessment was conducted to examine the blood supply to the follicle once it reached a size of either 15-16 mm or 17-18 mm. The administration of hCG injection was based on the Doppler findings, and the ensuing procedures were comparable to those in the TVS group.

Statistical analysis

The data analysis was performed using the IBM SPSS Statistics for Windows, Version 10.5 (Released 2000; IBM Corp., Armonk, New York, United States). The statistical significance between the outcomes of both groups was assessed using the student’s t-test, Mann-Whitney test, and Pearson's Chi-square test. A p-value below 0.05 was deemed statistically significant [13-15].

## Results

The comparative investigation of 64 women undergoing infertility treatment showed comparable age group distributions between the TVS and Doppler groups. Out of the patients who were 25 years old or older, 45.2% were in the TVS group and 47.1% were in the Doppler group. The total distribution was 46.2%. Within the age range of 26-30 years, 49.0% of individuals belonged to the TVS group, while 46.2% belonged to the Doppler group (Table 1). This resulted in an overall distribution of 47.6%. The TVS group had 5.8% of patients aged 31-35 years, while the Doppler group had 6.7%. Together, these two groups accounted for a total of 6.2% of the patients.

**Table 1:** Distribution of patients across different age groups TVS: transvaginal ultrasonography; N: number

Age (Yrs)	Group		Total N (%)
	TVS N (%)	Doppler N (%)	
>=25	14 (43.8%)	16 (50.0%)	30 (46.9%)
26-30	16 (50.0%)	15 (46.9%)	31 (48.4%)
31-35	2 (6.2%)	1 (3.1%)	3 (4.7%)
Total	32 (100%)	32 (100%)	64 (100%)

The results reveal that the distribution of patients across different age groups was similar in both the TVS and Doppler study groups, indicating that age was evenly distributed among the participants.

Intriguing patterns were identified when age categories and ovulation status were compared in the TVS and Doppler study cohorts. A total of 43.8% of patients in the TVS group who were 25 years of age or older ovulated, whereas 45.9% did not ovulate. On the contrary, an equivalent proportion of 47.1% of patients in the Doppler group failed to ovulate, while 47.1% ovulated.

A greater proportion of patients, 51.6%, in the TVS group (aged 26-30 years) ovulated, in contrast to the 47.9% who failed to ovulate. Within the same age cohort, 47.1% of the Doppler group ovulated, while 45.7% did not. Ovulation was observed in 6.2% of patients aged 31-35 years in the TVS group, while a mere 4.7% ovulated. Likewise, 5.7% of the Doppler group exhibited ovulation, while 7.1% failed to do so. The findings indicate that there are discrepancies in ovulation rates among various age groups, with marginally distinct trends identified in the TVS and Doppler study cohorts. Additional investigations will be undertaken to examine the importance of these disparities and their ramifications for the prediction of ovulation and the outcomes of fertility treatments (Table 2).

**Table 2:** Comparison between age and ovulation in TVS and Doppler TVS: transvaginal ultrasonography; N: number

Age (Yrs)	TVS		Doppler	
	TVS ovulated N (%)	TVS not ovulated N (%)	Doppler ovulated N (%)	Doppler not ovulated N (%)
>=25	14 (43.8%)	11 (45.9%)	16 (47.1%)	8 (47.1%)
26-30	17 (51.6%)	11 (47.9%)	16 (47.1%)	9 (45.7%)
31-35	2 (4.7%)	2 (6.2%)	2 (5.7%)	3 (7.1%)

Table 3 presents a comparison of ovulation status by BMI within the TVS and Doppler study groups. The data reveals that among patients with a normal BMI, the percentage of ovulation was slightly higher in the TVS group (34.4%) compared to the Doppler group (31.4%). Conversely, the percentage of patients who did not ovulate was higher in the Doppler group (36.4%) compared to the TVS group (40.4%). In the overweight category, a higher percentage of patients ovulated in both the TVS group (65.6%) and the Doppler group (68.6%) compared to those who did not ovulate. The table underscores the influence of BMI on ovulation status and suggests potential differences in ovulation rates between the TVS and Doppler study cohorts across different BMI categories. Further analysis is warranted to explore the implications of these findings for fertility treatment outcomes.

**Table 3:** Comparison of ovulation status by BMI in TVS and Doppler study groups TVS: transvaginal ultrasonography; BMI: body mass Index; N: number

BMI	TVS		Doppler	
	TVS ovulated N (%)	TVS not ovulated N (%)	Doppler ovulated N (%)	Doppler not ovulated N (%)
Normal (18.5-24.9)	11 (34.4%)	13 (40.4%)	11 (31.4%)	12 (36.4%)
Overweight (25-29.9)	21 (65.6%)	19 (59.6%)	24 (68.6%)	21 (63.6%)
Obese (>=30)	0	0	0	0

Table 4 displays the distribution of patients according to the type of infertility within both the TVS and Doppler study groups, as well as the total percentages. In the primary infertility subgroup, 50.0% of patients were included in the TVS group, while 47.1% were in the Doppler group, contributing to a total of 48.6% across both groups. For patients with secondary infertility, 50.0% were allocated to the TVS group, whereas 52.9% were assigned to the Doppler group, resulting in a total of 51.4% across both groups. These findings suggest a relatively balanced distribution of patients between the TVS and Doppler study groups across both primary and secondary infertility categories. Statistical analyses, such as Chi-square tests, can further elucidate any significant differences in distribution between the two groups and their implications for the study outcomes.

**Table 4:** Distribution according to type of infertility TVS: transvaginal ultrasonography; N: number

Infertility	Group		Total N (%)
	TVS N (%)	Doppler N (%)	
Primary	16 (50.0%)	16 (47.1%)	31 (48.6%)
Secondary	16 (50.0%)	18 (52.9%)	33 (51.4%)

The results in Table 5 illustrate the comparison of the diameter of follicles observed via TVS with ovulation status. In the TVS group, among follicles with a diameter of 15-16 mm, 11.4% of patients ovulated, while 29.5% did not ovulate. Similarly, for follicles with a diameter of 17-18 mm, 19.0% of patients ovulated, and 40.0% did not ovulate. Overall, out of the total sample size, 30.5% of patients in the TVS group ovulated, while 69.5% did not ovulate. The p-value of 0.501 indicates the level of statistical significance for the comparison. With a p-value greater than 0.05, there is no statistically significant difference in ovulation rates between follicles with diameters of 15-16 mm and 17-18 mm in the TVS group.

**Table 5:** Comparison of the diameter of the follicle in TVS with ovulation TVS: transvaginal ultrasonography; N: number The p-value indicates the statistical significance of the association between the diameter of the follicle and ovulation status observed through TVS. In this case, a p-value of 0.05 suggests a significant association between these variables, as it is less than the conventional significance level of 0.05

Group	Diameter of follicle	Ovulation		Total N (%)	p-value
		Ovulated N (%)	Not ovulated N (%)		
TVS	15-16 mm	24 (11.4%)	62 (29.5%)	86 (41.0%)	0.05
	17-18 mm	40 (19.0%)	84 (40.0%)	124 (59%)	
	Total	64 (30.5%)	146 (69.5%)	210 (100%)	

Table 6 compares the perifollicular blood flow peak systolic velocity (PSV) with ovulation. The data revealed a significant association between PSV values and ovulation. Specifically, as PSV increased, the likelihood of ovulation also increased significantly. This finding suggests that perifollicular blood flow, as measured by PSV, may serve as a valuable predictor of ovulation in women undergoing fertility treatment. In 64 patients, 58 cycles had a PSV of ≤9 cm/s, 16 cycles had a PSV of 9-10 cm/s, and 136 cycles had a PSV of >10 cm/s. Among the cycles with a PSV of ≤9 cm/s, eight resulted in ovulation, while 50 did not. For cycles with a PSV of 9-10 cm/s, 12 resulted in ovulation and four did not. In cycles with a PSV greater than 10 cm/s, 50 resulted in ovulation, while 86 did not.

**Table 6:** Comparison of perifollicular blood flow PSV in diameter of follicle with ovulation PSV: peak systolic velocity; N: number

PSV	Ovulation		Total N (%)
	Ovulated N (%)	Not ovulated N (%)	
<=9	8 (3.8%)	50 (23.8%)	58 (27.6%)
9-10	12 (5.7%)	4 (1.9%)	16 (7.6%)
>10	50 (23.8%)	86 (41.0%)	136 (64.8%)
Total	70 (33.3%)	140 (66.7%)	-

In Table 7, the perifollicular blood flow resistance index (RI) was examined in relation to ovulation. The results showed that RI values below 0.50 cm/s were associated with a higher rate of ovulation compared to higher RI values. This indicates that lower RI values may indicate better perifollicular blood flow, which in turn, may facilitate ovulation. Therefore, RI measurement could potentially serve as a useful tool in assessing ovarian function and predicting ovulation in infertility patients. In 64 patients, 80 cycles had RI between 0.53 and 0.56 cm/s, 46 cycles had RI between 0.50 and 0.53 cm/s, and 84 cycles had RI less than 0.50 cm/s. Among cycles with RI between 0.53 and 0.56 cm/s, 12 ovulated and 68 did not. For cycles with RI between 0.50 and 0.53 cm/s, 16 ovulated and 30 did not. In cycles with RI less than 0.50 cm/s, 42 resulted in ovulation and 42 did not.

**Table 7:** Comparison of perifollicular blood flow RI with ovulation RI: resistance index; N: number

RI	Ovulation		Total N (%)
	Ovulated N (%)	Not ovulated N (%)	
0.53-0.56	12 (5.7%)	68 (32.4%)	80 (38.1%)
0.50-0.53	16 (7.6%)	30 (14.3%)	46 (21.9%)
<0.50	42 (20.0%)	42 (20.0%)	84 (40.0%)
Total	70 (33.3%)	140 (66.7%)	-

Table 8 explored the association between endometrial vascularity and ovulation. The data revealed that higher vascularity, particularly in Zone 4, correlated with increased rates of ovulation. This suggests that endometrial vascularity may play a crucial role in the ovulatory process, possibly by providing a favourable environment for implantation and pregnancy. Therefore, assessing endometrial vascularity could be beneficial in predicting ovulation and optimising fertility treatment outcomes. In 64 patients, 11 cycles were in Zone 1, 31 cycles were in Zone 2, 70 cycles were in Zone 3, and 98 cycles were in Zone 4. Among cycles in Zone 1, none ovulated and 11 did not ovulate. In Zone 2, two ovulated and 29 did not ovulate. In Zone 3, 26 cycles resulted in ovulation while 44 did not. In Zone 4, 42 cycles resulted in ovulation while 56 did not.

**Table 8:** Comparison of endometrial vascularity with ovulation N: number

Endometrial vascularity	Ovulation		Total N (%)
	Ovulated N (%)	Not ovulated N (%)	
Zone 1	0 (0%)	11 (5.2%)	11 (5.2%)
Zone 2	2 (1.0%)	29 (13.8%)	31 (14.2%)
Zone 3	26 (12.4%)	44 (21.0%)	70 (33.3%)
Zone 4	42 (20.0%)	56 (26.7%)	98 (46.7%)
Total	70 (33.3%)	140 (66.7%)	-

In Table 9, the relationship between follicle diameter and pregnancy was investigated. While there was a higher conception rate among cycles with a diameter of 17-18 mm compared to 15-16 mm, the difference was not statistically significant. This suggests that follicle diameter alone may not be a reliable predictor of pregnancy outcomes. Out of 64 patients, 86 cycles had a follicle diameter of 15-16 mm, and 124 cycles had a follicle diameter of 17-18 mm. Among cycles with a follicle diameter of 15-16 mm, nine (4.3%) conceived and 77 (36.7%) did not conceive. For cycles with a follicle diameter of 17-18 mm, 19 (9.0%) conceived and 105 (50.0%) did not conceive. The statistical analysis revealed a p-value of 0.309, indicating that the difference in pregnancy rates between the two diameter groups is not statistically significant. It is essential to consider other factors, such as hormonal profiles and endometrial receptivity, in conjunction with follicle diameter when assessing fertility potential.

**Table 9:** Comparison between diameter of follicle and pregnancy N: number p-value = 0.309 (statistically not significant)

Diameter of follicle	Pregnancy		Total N (%)
	Conceived N (%)	Not conceived N (%)	
15-16	9 (4.3%)	77 (36.7%)	86 (41.0%)
17-18	19 (9.0%)	105 (50.0%)	124 (59%)
Total	28 (13.3%)	182 (86.7%)	-

Table 10 illustrates the impact of PSV on pregnancy outcomes. The data demonstrated a significant association between PSV and pregnancy, with higher PSV values correlating with increased conception rates. This finding highlights the importance of assessing perifollicular blood flow dynamics, as measured by PSV, in predicting pregnancy outcomes in infertility patients. Additionally, among the 64 patients, 58 cycles had a PSV of ≤9 cm/s, 16 cycles had a PSV of 9-10 cm/s, and 136 cycles had a PSV of >10 cm/s. Among the cycles with a PSV of ≤9 cm/s, five (2.4%) resulted in conception, while 53 (24.6%) did not result in conception. For cycles with a PSV of 9-10 cm/s, six (2.9%) resulted in conception, and 10 (4.8%) did not result in conception. In cycles with a PSV of >10 cm/s, 24 (11.4%) resulted in conception, while 112 (53.3%) did not result in conception. It suggests that interventions aimed at improving perifollicular blood flow may enhance fertility treatment success rates.

**Table 10:** Comparison between PSV and pregnancy DF PSV: Doppler flow peak systolic velocity; N: number The statistical analysis revealed a p-value of 0.020, indicating that there is a statistically significant difference in pregnancy rates between the PSV groups

DF PSV	Pregnancy		Total N (%)
	Conceived N (%)	Not conceived N (%)	
<=9	5 (2.4%)	53 (25.4%)	58 (27.6%)
9-10	6 (2.9%)	10 (4.8%)	16 (7.6%)
>10	24 (11.4%)	112 (53.3%)	136 (64.8%)
Total	35 (16.7%)	175 (83.3%)	-

Table 11 evaluated the influence of RI on pregnancy. The results indicated that lower RI values were associated with higher conception rates. This suggests that RI measurement may serve as a valuable tool in predicting ovulation and optimising fertility treatment outcomes. In 64 patients, 80 cycles had RI between 0.53 and 0.56 cm/s, 46 cycles had RI between 0.50 and 0.53 cm/s, and 84 cycles had RI less than 0.50 cm/s. Among cycles with RI between 0.53 and 0.56 cm/s, seven (3.3%) resulted in conception, while 73 (34.8%) did not result in conception. For cycles with RI between 0.50 and 0.53 cm/s, 10 (4.8%) resulted in conception, and 36 (17.1%) did not result in conception. In cycles with RI less than 0.50 cm/s, 18 (8.6%) resulted in conception, and 66 (31.4%) did not result in conception. Furthermore, it underscores the importance of assessing ovarian blood flow dynamics in infertility patients to identify those at higher risk of ovulatory dysfunction and poor treatment outcomes.

**Table 11:** Comparison between resistance index and pregnancy DF RI: Doppler flow resistance index; N: number The statistical analysis yielded a p-value of 0.05, indicating that there is a statistically significant difference in pregnancy rates between the RI groups

DF RI	Pregnancy		Total N (%)
	Conceived N (%)	Not conceived N (%)	
0.53-0.56	7 (3.3%)	73 (34.8%)	80 (38.1%)
0.50-0.53	10 (4.8%)	36 (17.1%)	46 (21.9%)
<0.50	18 (8.6%)	66 (31.4%)	84 (40.0%)
Total	35 (16.7%)	175 (83.3%)	-

Table 12 examines the relationship between endometrial vascularity and pregnancy. The findings revealed that higher vascularity, particularly in Zone 4, was significantly associated with increased rates of conception. This underscores the importance of endometrial receptivity in achieving successful pregnancy. In 64 patients, there were 11 cycles in Zone 1, 31 cycles in Zone 2, 70 cycles in Zone 3, and 98 cycles in Zone 4. Among cycles in Zone 1, none resulted in conception, and 11 (5.2%) did not result in conception. In Zone 2, one (0.5%) resulted in conception, and 30 (14.3%) did not result in conception. For cycles in Zone 3, 14 (6.7%) resulted in conception, while 56 (26.7%) did not result in conception. In Zone 4, 20 (9.5%) resulted in conception, while 78 (37.1%) did not result in conception. These outcomes highlight the potential utility of assessing endometrial vascularity as a predictor of fertility treatment success.

**Table 12:** Comparison between endometrial vascularity and pregnancy N: number The statistical analysis showed a p-value of 0.05, indicating that there is a statistically significant difference in pregnancy rates among different zones of endometrial vascularity

Endometrial vascularity	Pregnancy		Total N (%)
	Conceived N (%)	Not conceived N (%)	
Zone 1	0 (0%)	11 (5.2%)	11 (5.2%)
Zone 2	1 (0.5%)	30 (14.3%)	31 (14.8%)
Zone 3	14 (6.7%)	56 (26.7%)	70 (33.3%)
Zone 4	20 (9.5%)	78 (37.1%)	98 (46.7%)
Total	35 (16.7%)	175 (83.3%)	-

## Discussion

The development of high-resolution ultrasonography has significantly transformed the area of assisted reproduction by enabling the detection of uterine or ovarian sonographic markers that can forecast treatment outcomes [12,15]. This study involved the analysis of several ultrasonographic factors, such as age, parity, BMI, TVS for ovulation assessment, and vascular changes found in Doppler investigations. The aim was to determine the relationship between these parameters and the pregnancy outcomes in assisted reproduction therapy cycles. The findings on age, parity, and BMI are consistent with other studies, such as the research conducted by Cogendez et al. [6], which similarly found no significant variations in these factors among the participants. These findings indicate that factors such as age, parity, and BMI do not have a separate impact on pregnancy outcomes in assisted reproduction therapy cycles. Our study aimed to examine the utilisation of TVS in identifying ovulation. This is essential to guarantee the effectiveness of fertility drugs. TVS offers a precise and comprehensive visualisation of follicle development, facilitating the distinction between mature follicles and luteinised, unruptured follicles or polycystic ovaries [10]. Although prior research, such as the study conducted by Majeed et al. [7], has emphasised the efficacy of utilising ultrasonographic periovulatory follicular tracking for predicting ovulation, our investigation did not discover a statistically significant association between follicle width and ovulation. Subsequently, we conducted an analysis of alterations in blood vessels utilising Doppler ultrasound, with a specific focus on evaluating the characteristics of blood flow in the area surrounding the hair follicles. Doppler examinations have played a crucial role in assessing the blood flow in the uterine and ovarian arteries. The PSV is utilised as a quantitative indicator of the likelihood of oocyte formation. The findings of our analysis support the results of previous studies conducted by Mayer et al. [11] and van Duijn et al. [9], which showed a favourable correlation between PSV levels surpassing 10 cm/s and increased rates of ovulation and conception. This suggests that PSV has the capacity to function as a valuable tool for predicting the outcome of a treatment. In addition, our analysis examined the RI values, where an RI < 0.50 cm/s suggests a higher probability of fertility. This discovery supports prior studies, indicating that evaluating RI can be a useful predictor for ovulation and treatment results. In addition, we evaluated the blood flow in the endometrium using Doppler ultrasound, with specific attention to various regions inside the endometrium. The results of our investigation confirmed the findings of previous studies conducted by Ahmadi et al. [10] and Smart et al. [5]. The findings revealed a direct correlation between enhanced blood vessel formation in Zones 3 and 4 of the endometrium and higher rates of ovulation and pregnancy [14,16]. This highlights the need to evaluate the responsiveness of the uterus and predict treatment outcomes by taking into account levels of vascularity.

Work limitations

Although our work offers useful insights into the predictive significance of ultrasonographic parameters in assisted reproduction therapy cycles, it is not exempt from limitations. Initially, the size of the sample in our study may not be adequate to fully encompass the range of variability within the group receiving assisted reproduction therapy. Furthermore, our study specifically examined ultrasonographic data and did not consider other potential variables that could affect treatment outcomes, such as lifestyle factors or underlying medical disorders. Moreover, the inherent retrospective nature of our study design may potentially add bias to our findings. This is because the data-gathering process depended solely on medical records, which may not consistently provide comprehensive or precise information. Additional research using bigger sample sizes and prospective study designs is necessary to have a better understanding of how ultrasonographic characteristics can predict treatment success in assisted reproductive therapy cycles.

## Conclusions

The implementation of high-resolution ultrasonography has revolutionised assisted reproduction by enabling the identification of uterine or ovarian sonographic markers predictive of treatment outcomes. Our study aimed to establish correlations between various ultrasonographic parameters and pregnancy outcomes in assisted reproduction therapy cycles. The results demonstrated that age, parity, and BMI do not independently influence pregnancy outcomes, aligning with findings from previous studies. TVS effectively detected ovulation, providing a clear view of follicle growth, although no significant correlation between follicle diameter and ovulation was found. Doppler ultrasound assessments of PSV and RI proved valuable in predicting ovulation and conception, with higher PSV and lower RI values indicating better blood supply and increased likelihood of successful outcomes. These findings suggest that ultrasonographic measures, particularly TVS and Doppler assessments of blood flow dynamics, are crucial for predicting pregnancy outcomes in assisted reproduction therapy. Further research is necessary to validate these findings and explore additional factors impacting treatment efficacy, ultimately contributing to improved fertility treatment strategies and patient outcomes.
